# Mendelian Randomization–Based Analysis of Causal Effects Between Statins and Common Upper Gastrointestinal Diseases

**DOI:** 10.1155/grp/5335710

**Published:** 2026-10-29

**Authors:** Wenchu Zhao, Jiejun Hou, Jinyi Chen, Jinhua Pang

**Affiliations:** ^1^ Shaanxi University of Chinese Medicine Xianyang China sntcm.edu.cn; ^2^ Shaanxi University of Traditional Chinese Medicine Hospital Xianyang China

**Keywords:** diseases, *Helicobacter pylori*, Mendelian randomization, statins, upper gastrointestinal

## Abstract

**Background:**

Statins are widely used for cardiovascular disease prevention and exhibit multiple pleiotropic effects, including anti‐inflammatory properties. However, conclusions from observational studies regarding the causal relationship between statin use and upper gastrointestinal diseases (such as gastric cancer, gastric ulcer, and functional dyspepsia) remain controversial. This study is aimed at systematically evaluating the causal association between statin‐targeted gene (HMGCR)–mediated reductions in low‐density lipoprotein cholesterol (LDL‐C) levels and common upper gastrointestinal diseases using drug‐targeted Mendelian randomization (MR). This approach seeks to uncover potential unintended effects.

**Methods:**

We extracted genetic variants associated with the HMGCR gene region from genome‐wide association studies (GWAS) as instrumental variables to simulate the lipid‐lowering effects of statins. Outcome data were derived from large‐scale GWAS meta‐analyses covering gastric cancer (*n* = 202,308), gastroenteritis (*n* = 7651), gastric ulcer (*n* = 361,194), gastroesophageal reflux disease (*n* = 462,933), and functional dyspepsia (*n* = 84,572). The primary analysis employed inverse variance weighting (IVW), with sensitivity assessments for heterogeneity and pleiotropy conducted via MR‐Egger, weighted median, and MR‐PRESSO methods.

**Results:**

Genetically predicted HMGCR‐mediated LDL‐C reduction was associated with reduced risk of gastric cancer (OR = 0.588, 95% CI: 0.423–0.816, *p* = 0.002), gastroenteritis (OR = 0.257, 95% CI: 0.086–0.766, *p* = 0.015), and gastric ulcer (OR = 0.996, 95% CI: 0.993–0.998, *p* = 0.003). However, analysis indicated a potential increased risk of functional dyspepsia (OR = 1.437, 95% CI: 1.014–2.036, *p* = 0.042). No significant causal association was found between statin use and gastroesophageal reflux disease (OR = 0.999, 95% CI: 0.992–1.006, *p* = 0.773). Sensitivity analyses confirmed the robustness of the results, with no evidence of significant level‐specific multiplicity.

**Conclusion:**

This study provides genetic evidence supporting the potential protective effects of statins against gastric cancer, gastritis, and gastric ulcers, which may be associated with their anti‐inflammatory properties. However, the potential increased risk of functional dyspepsia suggests the complexity of the drug′s effects. These findings provide scientific basis for the repositioning of statins in gastrointestinal diseases and their safety assessment.

## 1. Introduction

Statins (3‐hydroxy‐3‐methylglutaryl‐coenzyme A reductase inhibitors) are cornerstone medications for the clinical prevention and treatment of atherosclerotic cardiovascular disease. Beyond lowering low‐density lipoprotein cholesterol (LDL‐C) levels by inhibiting HMG‐CoA reductase [1], statins exhibit broad “multifunctionality,” including anti‐inflammatory, immunomodulatory, and antioxidant properties [2]. Given the pivotal role of chronic inflammation and dyslipidemia in the pathogenesis of various gastrointestinal disorders, the potential effects of statins on the gastrointestinal tract have garnered significant attention in recent years [3, 4].

Previous observational studies and meta‐analyses suggest that statin use may reduce the risk of chronic gastritis and gastric cancer, and may inhibit the development of peptic ulcers [5, 6]. Potential mechanisms may involve suppressing the release of proinflammatory cytokines (such as TNF‐*α* and IL‐6) and modulating *Helicobacter pylori*–induced inflammatory responses [7]. However, existing evidence remains controversial. Some studies indicate no association between statins and reduced risks of peptic ulcers or reflux esophagitis. Animal experiments even suggest statins may be ineffective in chemopreventing gastric cancer [8]. Furthermore, systematic evaluations regarding whether statins increase the risk of nonorganic diseases such as functional dyspepsia are currently lacking. These inconsistent conclusions may be attributed to unavoidable confounding factors in traditional observational studies (e.g., lifestyle and concomitant medications) and reverse causality bias.

To overcome the limitations of traditional epidemiological studies, Mendelian randomization (MR) has emerged as an innovative epidemiological approach. It utilizes genetic variation—primarily single nucleotide polymorphisms (SNPs)—as instrumental variables to infer causal relationships between exposures and outcomes [9]. Because alleles are randomly assigned during meiosis and remain fixed thereafter, MR studies are conceptually analogous to “naturally occurring randomized controlled trials,” effectively reducing confounding biases and reverse causality interference [10]. Specifically, drug‐target MR analysis employs genetic variants within specific drug‐target genes (e.g., HMGCR) as instrumental variables. The rigorous application of this causal inference framework is further supported by several classic methodological studies. These foundational literatures systematically elucidate the standardized procedures for valid instrumental variable selection, rigorous evaluation of horizontal pleiotropy, and robustness checks. By adhering to these established methodological paradigms, drug‐target MR effectively simulates the long‐term, on‐target biological effects of pharmacological interventions, thereby providing highly reliable genetic evidence for assessing both drug repurposing opportunities and long‐term safety profiles [11, 12]. This approach simulates the long‐term biological effects following inhibition of a particular drug target, thereby evaluating a drug′s potential for repurposing and its safety for prolonged use [13].

To date, no study has systematically evaluated the causal association between statins and a range of common upper gastrointestinal diseases (including gastric cancer, gastroenteritis, gastric ulcer, gastroesophageal reflux disease, and functional dyspepsia) using drug‐targeted MR methods. Therefore, this study is aimed at investigating the causal effects of statins on upper gastrointestinal diseases by simulating HMGCR inhibition‐mediated LDL‐C reduction, based on large‐scale genome‐wide association study (GWAS) data. This approach not only helps clarify the unintended indications of statins in the digestive system but also provides a genetic basis for their clinical risk management.

### 1.1. Instrumental Variable Data Source

The GWAS data for LDL‐C in the MR analysis were sourced from the UK Biobank data released in 2022, comprising 201,678 samples and 12,321,875 SNPs. UK Biobank is a large‐scale biomedical database and research resource. Since 2006, it has collected biological and medical data from over 500,000 individuals as part of a large‐scale prospective study. Participants in the biobank are aged between 40 and 69 years, with an average age of 56.5 years. Ninety‐four percent self‐reported European ancestry, and 54.4% were female. Biological samples such as blood, urine, and saliva are regularly collected from participants, alongside detailed information about their social lives and ongoing health monitoring.

Statins inhibit HMG‐CoA reductase, thereby reducing the synthesis of LDL‐C [14, 15]. Low‐density lipoprotein is a carrier protein whose functions include transporting cholesterol from blood vessels to peripheral tissue cells. LDL can enter the coronary artery endothelium, oxidize into oxidized LDL, and be phagocytosed by macrophages. Due to its high cholesterol content, LDL leads to substantial cholesterol deposition within the local vascular wall, gradually forming atherosclerotic plaques that cause coronary artery ischemia and hypoxia [16, 17]. Therefore, we selected LDL‐C as the instrumental variable.

### 1.2. Data Source for Final Outcome

Genetic data for gastroesophageal reflux disease, gastric ulcer, gastric cancer, gastritis, and functional dyspepsia were obtained from the IEU GWAS database. Data for gastritis, gastroesophageal reflux disease, and gastric ulcer were derived from the MRC‐IEU GWAS study. Detailed sample sizes and SNP counts are presented in Table 1.

**Table 1:** Genetic data of the disease.

Outcomes(disease)	Sample	SNP
Gastric cancer	202,308	8,885,324
Gastric reflux	462,933	9,851,867
Gastroenteritis	7651	9,807,446
Gastric ulcer	361,194	10,452,088
Functional dyspepsia	84,572	16,380,380

### 1.3. Instrumental Variable Screening

In this study, the instrumental variables selected were the common pharmacological target genes (HMGCR, HDAC2, and ITGAL) shared by statins (including lovastatin, simvastatin, and pravastatin) frequently used in clinical practice both domestically and internationally. The pharmacologic target genes encoding these drugs were identified in the DrugBank database, with detailed gene information retrieved from the GEO database. Table 2 presents the genomic region (GRCh37.p13), chromosome (CHr) number, and location of the selected drug target genes (this study primarily focuses on HMGCR inhibitors).

**Table 2:** Genetic information.

Drug	Drug target genes	Chromosome numbers	Genomic regions
Statins			
HMGCR inhibitors	HMGCR	Chr5	74,632,993–74,657,941
	HDAC2	Chr6	114,254,192–114,292,312
	ITGAL	Chr16	30,484,063–30,534,506

### 1.4. Research Design

We first extracted LDL‐C exposure data and removed linkage disequilibrium (LD). To capture more SNPs associated with upper gastrointestinal disease outcomes, we applied a threshold of *p* < 5 × 10^−8^. SNPs were aggregated using LD thresholds *r* ^2^ = 0.3 and kb = 100 to obtain independent genetic variants. Next, we read gene information to extract SNPs associated with drug targets. Subsequently, outcome data were extracted, and exposure and outcome datasets were merged. MR‐PRESSO outlier detection was performed, followed by MR analysis. The inverse variance weighting (IVW) method served as the primary approach, supplemented by heterogeneity analysis, pleiotropy testing, and leave‐one‐out sensitivity analysis. It is worth noting that although standard two‐sample MR practices typically recommend a strict LD clumping threshold (e.g., *r* ^2^ < 0.001) to ensure complete independence of instrumental variables, drug‐target MR analyses often face the challenge of having very few or no eligible SNPs within a highly restricted specific gene region (such as HMGCR). To balance the independence of instruments and the requirement for sufficient genetic variants to perform robust sensitivity analyses (e.g., pleiotropy assessment via MR‐Egger, which requires multiple SNPs), we relaxed the LD threshold to *r* ^2^ = 0.3 and kb = 100. This less stringent threshold has been widely adopted and validated in previous drug‐targeted MR studies to maintain adequate statistical power for comprehensive sensitivity testing. All statistical analyses were performed using R software (Version 4.3.2). MR analyses were conducted using the “TwoSampleMR” package, and outlier detection was performed using the “MRPRESSO” package (Figure 1).

**Figure 1:**
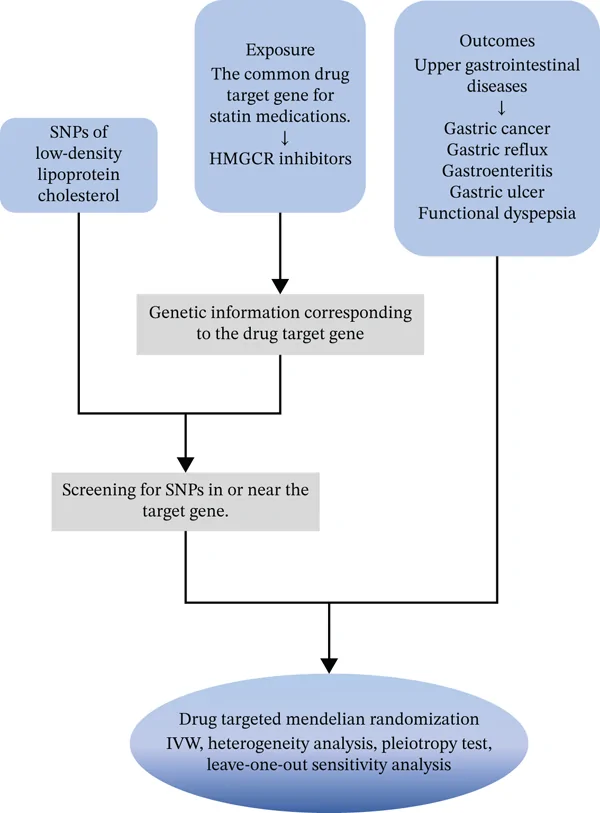
Experimental design diagram.

### 1.5. The Causal Relationship Between Blood Lipid Levels and Gastrointestinal Diseases

This study employed IVW, MR‐Egger, and weighted median methods for MR analysis. Simultaneous analysis of SNP heterogeneity and horizontal pleiotropy was conducted. Global tests were performed to validate the stability of results. Analysis of the relationship between LDL‐C and upper gastrointestinal diseases revealed that LDL‐C levels were associated with the risk of gastric cancer, gastritis, gastric ulcer, and functional dyspepsia (*p* < 0.05). Specifically, LDL‐C levels were positively associated with reduced risk of gastric cancer, gastritis, and gastric ulcer (OR < 1), whereas negatively associated with reduced risk of functional dyspepsia (OR > 1). This indicates that reduced LDL‐C levels exert a protective effect against gastric cancer, gastroenteritis, and gastric ulcers.

Specifically, LDL‐C levels were associated with a reduced risk of gastric cancer (OR = 0.587; *p* = 0.002); LDL‐C levels were associated with a reduced risk of gastroenteritis (OR = 0.257; *p* = 0.014); LDL‐C levels were associated with a reduced risk of gastric ulcer (OR = 0.996; *p* = 0.003); LDL‐C levels were associated with a reduced risk of functional dyspepsia (OR = 1.437; *p* = 0.041). No significant association was observed between LDL‐C levels and gastroesophageal reflux.

### 1.6. Genetic Specificity Analysis of the Causal Effects of Lipid‐Lowering Drugs on Gastrointestinal Diseases

The target genes of statins exhibit statistical significance in relation to gastric cancer, gastroesophageal reflux disease, gastroenteritis, and gastric ulcer. Specifically, the causal effect estimate of HMGCR‐mediated serum LDL‐C levels on gastric cancer is *β* = −0.532; the causal effect estimate of HMGCR‐mediated serum LDL‐C levels on gastroesophageal reflux disease was *β* = −0.001; the causal effect estimate of HMGCR‐mediated serum LDL‐C levels on gastroenteritis was *β* = −1.359; the causal effect estimate of HMGCR‐mediated serum LDL‐C levels on gastric ulcer was *β* = −0.004. However, no statistically significant association was found between statin target genes and functional dyspepsia; the causal effect estimate of HMGCR‐mediated serum LDL‐C levels on functional dyspepsia was *β* = 0.363 (Figure 2).

**Figure 2:**
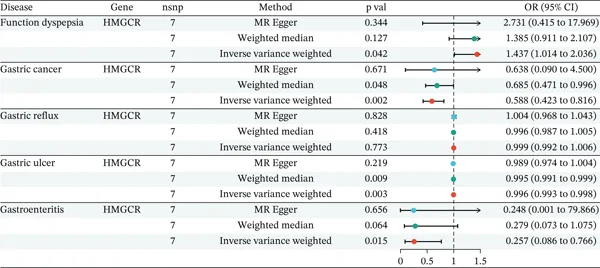
Genetic specificity results of the causal effects of lipid‐lowering drugs on gastrointestinal diseases. Bold values indicate statistical significance (*p* < 0.05).

This indicates that for every 1‐mmHg reduction in LDL‐C levels induced by HMGCR inhibitors, the risk of gastric cancer, gastroesophageal reflux disease, gastroenteritis, and gastric ulcer disease correspondingly decreases. Conversely, it suggests that for every 1 mmHg reduction in LDL‐C levels induced by HMGCR inhibitors, the risk of functional dyspepsia correspondingly increases.

### 1.7. Sensitivity Analysis

This study conducted sensitivity analysis using the leave‐one‐out method, as shown in Figure 3. The leave‐one‐out forest plot indicates no SNPs capable of altering the estimated causal effect.

**Figure 3:**
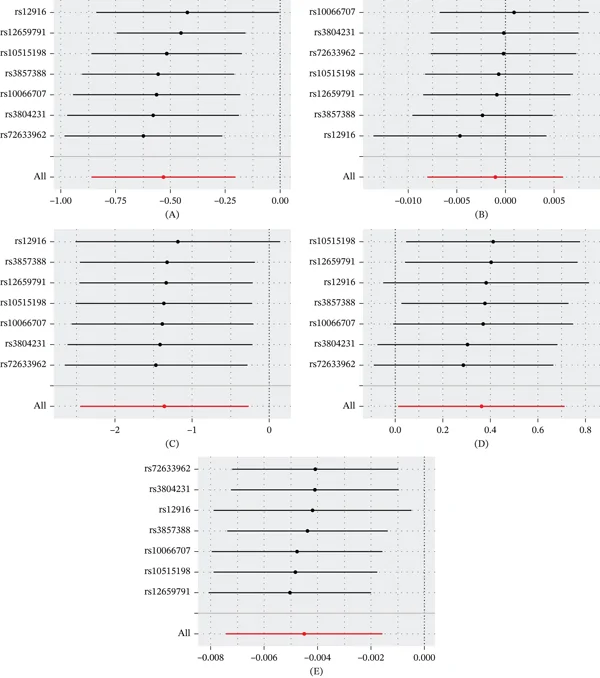
Leave‐one‐out method results of HMGCR inhibitors. (A) Gastric cancer. (B) Gastric reflux. (C) Gastroenteritis. (D) Gastric ulcer. (E) Functional dyspepsia.

Specifically, removing any single SNP does not result in significant differences between the effect values of the remaining SNPs and the pooled SNP effect value, further validating the reliability of the MR analysis results. The Cochran′s *Q* statistic for heterogeneity analysis was > 0.05, indicating no significant heterogeneity in the experimental data. Multifactoriality analysis revealed that multifactoriality occurs when instrumental variables influence outcomes through factors other than the exposure variable. An MR‐Egger regression intercept *p* > 0.05 indicates no multifactoriality, meaning horizontal multifactoriality does not bias the results. As shown in Table 3, neither significant heterogeneity nor multifactoriality was present in the study data.

**Table 3:** Heterogeneity and multifunctionality.

Drug	Outcomes	MR‐Egger regression	Horizontal pleiotropy
		Q_*p*val	P
HMGCR inhibitors	Gastric cancer	0.153	0.936
	Gastric reflux	0.373	0.785
	Gastroenteritis	0.994	0.991
	Stomach ulcers	0.760	0.442
	Functional dyspepsia	0.778	0.527

## 2. Discussion

The cytotoxins CagA and VacA produced by *H*. *pylori* exert toxic effects on gastric mucosal cells, inducing the production of proinflammatory cytokines (TNF‐*α*, IL‐1, and IL‐6). This process recruits macrophages and neutrophils to the infection site, triggering a survival response in gastric epithelial cells [18]. Subsequently, infection induces massive urease production while suppressing phagocyte activity and T cell function, thereby altering gastric physiology and disrupting gastrin secretion. This alteration reduces gastric mucosal hydrophobicity, directly disrupting epithelial cells to release inflammatory mediators. This triggers localized mucosal inflammation, leading to gastritis and potentially gastric cancer. It also induces excessive gastric acid secretion, damaging the normal mucosal barrier and severely progressing to gastric ulceration [19].

In the mechanism of statin‐mediated inhibition of the HMG‐CoA reductase pathway, alterations in statin uptake or metabolism also modify mitochondrial function, calcium signaling, and cell cycle pathways. Compounds affected by HMG‐CoA reductase inhibition include isoprenylated proteins, electron transport proteins, and heme A [20]. This can lead to downstream effects such as impaired cell membrane stability and excitability, disrupted signal transduction and intracellular transport, and compromised protein structure and function. Collectively, these alterations may result in dysfunction or reduction of membrane receptors, channels, and transporters, alongside decreased gene expression [21].

*H*. *pylori*–associated infection ranks among the most common triggers of human gastrointestinal diseases. This infection disrupts the mucosal barrier, ultimately causing inflammatory lesions in gastric mucosal cells. Statins enhance the efficacy of anti–*H*. *pylori* treatment, attributed to their anti‐inflammatory properties.

Statins suppress acute and chronic inflammation by interfering with endothelial adhesion molecules and the transendothelial migration of granulocytes to inflammatory sites. Furthermore, statins reduce activation of the monocyte/macrophage system, and this suppression of activation confers numerous beneficial effects on T lymphocytes [22, 23], including decreased T lymphocyte cytotoxicity. Proinflammatory helper T cell subsets are suppressed by statins. Statin‐induced cholesterol depletion promotes autophagy in *H*. *pylori*–containing macrophages [24], thereby reducing bacterial burden. Several studies have reported elevated serum CRP levels in patients with chronic *H*. *pylori* infection. Furthermore, associations between *H*. *pylori* gastritis and cardiovascular disease risk have been reported using inflammatory biomarkers, including LDL‐C [25]. However, another study failed to confirm a positive correlation between *H*. *pylori* infection, serum CRP levels, and coronary heart disease risk [26]. *H*. *pylori* is a gram‐negative microaerophilic spirochete colonizing the human stomach, estimated to infect over half the global population. Although commonly regarded as an intracellular pathogen, this organism resides within the mucosal layer and tightly adheres to the gastric epithelial surface. Notably, virulent strains of *H*. *pylori* can delay phagocytosis and promote the formation of intraluminal bodies within macrophages, a key feature of *H*. *pylori*–induced pathogenesis. Van Oijen et al. [27], through epidemiological investigations, found significantly lower vitamin B12 levels in a large cohort of cardiovascular disease patients. Adequate vitamin B12 absorption requires proper function of the gastric parietal mucosa. In the Netherlands, approximately one‐fifth of cardiovascular disease patients with severe vitamin B12 deficiency had chronic atrophic gastritis as a background condition, supporting the association between cardiovascular disease medications and upper gastrointestinal disorders.

Statins such as simvastatin not only reduce cellular cholesterol but also promote *H*. *pylori*–induced macrophage autophagy [28]. Statin therapy enhances *H*. *pylori*–induced autophagy, subsequently promoting autophagosome maturation and fusion with lysosomes, thereby reducing bacterial load in macrophages and improving inflammatory responses [29]. Statins may be utilized to reduce intracellular *H*. *pylori* load within macrophages. Furthermore, statins enhance early endosomal maturation and subsequent autophagy activation in macrophages, thereby promoting lysosomal fusion. This leads to the degradation of sequestered *H*. *pylori*, subsequently reducing proinflammatory cytokine production [30]. The role of statins in reducing Cag A protein‐induced inflammation caused by *H*. *pylori* in gastric mucosa by lowering cellular cholesterol levels and reducing IL‐8 has also been reported. Simvastatin promotes apoptosis in gastric cancer cells while inhibiting proliferation and metastasis. In gastric cancer–related studies, Liu et al. [31] concluded that simvastatin promotes apoptosis and inhibits proliferation and metastasis in gastric cancer cells. The mechanism involves statins inhibiting HMG‐CoA reductase, downregulating geranylgeranyl pyrophosphate, thereby reducing RhoA activity levels and suppressing YAP and *β*‐catenin activity. Therefore, integrating prior studies, our MR analysis indicates that statin‐targeted genes confer protective effects against gastrointestinal cancer risk reduction (*β* = −1.359). Caldas et al. [32] also revealed an association between statin use and the efficacy of bismuth‐based quadruple therapy. Cheung et al. [33] concluded in a propensity score‐matched study on gastric cancer that long‐term statin use, in a dose‐ and duration‐dependent manner, was associated with lower gastric cancer risk in *H*. *pylori*–eradicated patients. This study further supports the evidence that statins offer additional benefits as immunomodulatory agents against gastric cancer in *H*. *pylori*–eradicated individuals.

Interestingly, our analysis revealed a divergent effect of statins: Although protective against organic lesions (gastric cancer, ulcers, and gastritis), genetically predicted HMGCR inhibition was associated with increased functional dyspepsia risk (OR = 1.437). This finding warrants cautious interpretation. Unlike organic diseases triggered by inflammation or *H*. *pylori* infection, functional dyspepsia typically manifests as abnormal gastric motility, visceral hypersensitivity, and gut‐brain axis dysfunction.

We propose the following potential risk mechanism hypotheses: First, statins are known to affect muscle mitochondrial function, potentially leading to subclinical myopathy. A similar mechanism may act on gastric smooth muscle, causing delayed gastric emptying or adaptive dysfunction—both hallmarks of functional dyspepsia. Second, statins may induce nonulcerative mucosal irritation or alter the gastric microenvironment, enhancing visceral sensitivity without causing visible lesions. Third, given the lipophilic nature of certain statins and their ability to cross the blood–brain barrier, their effects on the central nervous system may operate via the gut–brain axis. These hypotheses require validation through complementary approaches such as phenotype‐associated genome‐wide studies (PheWAS) or observational cohorts with detailed symptom‐level data.

## 3. Innovation and Shortcomings

Our study possesses several strengths, including the following: First, we provide the first evidence of a causal relationship between serum LDL‐C and upper gastrointestinal diseases using GWAS data. Second, based on genetic data, we identified a protective effect of statins against gastric cancer, gastroenteritis, and gastric ulcers. Third, we employed multiple methods to validate MR results, including estimating *F*‐statistics to exclude weak instrumental variables and concurrently using IVW and MR‐PRESSO outlier detection to verify accuracy, thereby minimizing confounding biases and reverse causality.

However, this study also has limitations.

First, it primarily relies on pooled GWAS statistics from populations of European ancestry, mainly sourced from the UK Biobank. This limits the generalizability of findings to other populations. This limitation is particularly pronounced in gastric cancer research, as East Asian populations exhibit significantly higher gastric cancer incidence than Western populations. Therefore, we strongly recommend future studies validate results using large‐scale East Asian or multiethnic cohorts, such as the Japanese Biobank or the Chinese Kadoorie Biobank, to confirm the external validity of the association between statins and upper gastrointestinal diseases.

Second, despite stringent selection criteria, the number of SNPs available after LD pruning remained relatively small. Although the *F*‐statistics for the selected instrumental variables demonstrated sufficient power (*F* > 10) to effectively limit weak instrumental bias, statistical power may still be constrained. Future studies could further explore these causal associations with enhanced statistical power by employing polygenic risk scores (PRS) for LDL‐C or leveraging specific statin response variants.

Third, we employed genetic variants in the HMGCR gene as a proxy for statin treatment exposure. Although this approach effectively assesses lifetime exposure to LDL‐C lowering, it cannot fully capture real‐world pharmacologic complexities such as differences between hydrophilic and lipophilic statins, dose–response relationships, or medication adherence. Furthermore, statins may exhibit pleiotropic effects unrelated to LDL‐C reduction. Future studies integrating pharmacoepidemiological data or conducting MR analyses of drug exposure using pharmacogenomic variants known to influence statin metabolism (e.g., SLCO1B1 gene) could contribute to a more comprehensive understanding.

Fourth, we must acknowledge the potential limitation of sample overlap between our exposure and outcome datasets. The LDL‐C GWAS was derived from the UK Biobank, and several outcome GWASs (e.g., gastric ulcer, gastroesophageal reflux disease, and gastritis) obtained from the MRC‐IEU also include substantial UK Biobank cohorts. In two‐sample MR, sample overlap can bias causal estimates towards observational associations and inflate Type I error rates, particularly when instruments are weak. Although we calculated the *F*‐statistics for all selected SNPs (*F* > 10), indicating strong instruments that can substantially mitigate the bias caused by sample overlap, this residual risk cannot be entirely eliminated. Future studies utilizing completely independent large‐scale cohorts (e.g., FinnGen) for outcome assessment are required to further validate our findings.

## Funding

No funding was received for this manuscript.

## Disclosure

The authors take full responsibility for all machine‐generated content to ensure its scientific accuracy and appropriateness.

## Conflicts of Interest

The authors declare no conflicts of interest.

## Supporting Information

Additional supporting information can be found online in the Supporting Information section.

## Supporting information


**Supporting Information 1**  

**Supporting Information 2** File S1: STROBE‐MR checklist.

## Data Availability

The data that support the findings of this study are available on request from the corresponding author. The data are not publicly available due to privacy or ethical restrictions.
