# Supplementary material for: Mendelian Randomization–Based Analysis of Causal Effects Between Statins and Common Upper Gastrointestinal Diseases
Source: Gastroenterol Res Pract. 2026 Oct 29;2026:5335710. doi: 10.1155/grp/5335710 (PMC13717593; doi:10.1155/grp/5335710)
Supplement: Supplementary file 2 [file GRP-2026-5335710-s002.docx]

We used Mendelian randomization to investigated the causal relationship between common upper gastrointestinal diseases and HMGCR related SNPs targeted by statin drugs that regulate LDL-C levels. The IVW results showed that serum LDL-C levels mediated by HMGCR were positively correlated with the risk of gastric cancer, gastroenteritis, and gastric ulcer. Therefore, our study suggests a causal relationship between statins as HMGCR inhibitors and gastric cancer, gastroenteritis, and gastric ulcers. HMGCR expression inhibits acute and chronic inflammation and reduces activation of mononuclear or macrophage systems.
